# Student teachers’ perceived changes of learning conditions during COVID-19: The role of internal resource management strategies, intrinsic motivation, and preferences for lesson formats

**DOI:** 10.3389/fpsyg.2022.894431

**Published:** 2022-08-12

**Authors:** Jennifer Paetsch, Anne Schlosser

**Affiliations:** Institute for Educational Science, University of Bamberg, Bamberg, Germany

**Keywords:** COVID-19, self-regulated learning, resource-management strategies, higher education, intrinsic motivation, student teacher, emergency remote teaching

## Abstract

The COVID-19 pandemic caused an abrupt change in higher education that had a profound impact on students. Pandemic distance learning required students to regulate their learning more independently and to find new ways of communicating with their peers and instructors. This study focused on how students perceived the learning conditions that they encountered during the first semester that took place online compared to the time before distance learning. The primary aim of this study was to determine whether students’ internal resource management strategies, intrinsic motivation, and instruction format (synchronous, asynchronous, and face-to-face) preferences were associated with the perceived changes of the learning conditions. Students enrolled in a German university (*N* = 330) answered an online questionnaire at the end of the summer term in 2020. Findings from structural equation modeling showed that the regulative resources of attention and intrinsic motivation were significant factors that predicted how students perceived changes in relevance, quality, and support of online instruction compared to the time before distance learning. However, our results show that these factors did not impact perceived changes in social relatedness. Moreover, the results demonstrate that preferences for digital formats were significant related to student perceptions of changes in relevance, quality, and support, whereas preferences for the face-to-face format had significant negative effects on these factors. Only the face-to-face preference had a significant (negative) effect on social relatedness. Finally, the study revealed an indirect effect of attention on students’ perceived changes of learning conditions through preferences for lesson formats. This study has important implications for digital integration in higher education and suggests that institutions should implement various methods that foster social interaction and internal regulation strategies.

## Introduction

To restrict social contact during the COVID-19 pandemic, many countries implemented a range of measures, including the temporary closure of educational institutions and the widespread introduction of distance learning in schools, colleges, and universities (UNICEF, 2020). In higher education (HE), students experienced fundamental changes to their learning experience in just a matter of weeks, with most face-to-face courses being replaced by online education (Crawford et al., 2020; Mishra et al., 2020). HE institutions were closed in 185 countries worldwide (Marinoni et al., 2020), and, in Europe, 15% of HE institutions abandoned all teaching activities, while 85% replaced classroom teaching with remote instruction (Marinoni et al., 2020).

Overall, many creative digital solutions for distance or online teaching quickly emerged in response to the pandemic. In general, there are two different online learning formats: asynchronous online learning, which is independent of time and place, and synchronous online learning, which is independent of location but held at the same time.

The rapid switch to online learning settings (Crawford et al., 2020; Garcia-Morales et al., 2021) posed tremendous challenges to educators, universities, and students. Emergency remote teaching was characterized by a lack of time, skills, and infrastructure on the part of both institutions and university educators, who implemented their courses through virtual formats and ensured the continuation of lessons (Adedoyin and Soykan, 2020; Hodges et al., 2020). At the same time, higher levels of autonomy, self-regulation, and intrinsic motivation were required from students due to a lack of access to support systems, a disruption of routines, and reduced social interactions (Naujoks et al., 2021; Pelikan et al., 2021a).

The current study is situated in the outstanding situation of emergency remote teaching and learning. As emergency remote education is different from regular education, it is crucial to understand whether and how students experienced the new situation and to gain a deeper understanding of students’ perceptions of the learning situation. This study draws upon a larger amount of research that aims to understand how HE students perceived their online teaching and learning experiences and adapted to the situation (e.g., Cavanaugh et al., 2022; Corpus et al., 2022; Lobos et al., 2022). Understanding the challenges that students face while learning remotely will enable HE institutions and university educators to develop strategies and structures, which can assist students to learn more effectively in online learning environments. If HE institutions know how their students perceived the offered learning environment during the pandemic as compared to the time before it, they may be able to adjust their services in the future and take individual differences in needs between students into account (Gruber et al., 2010).

In order to find out how students managed this situation, it is important to examine factors that predict students’ experiences and perceptions of the new digital teaching and learning formats. To our knowledge, there is a scarcity of empirical studies focused on students’ predispositions in this context. Specifically, the role of resource management strategies in adapting successfully to emergency remote education still needs to be clarified. Therefore, the primary aim of this study was to investigate how HE students perceived the learning conditions that they encountered during the first semester that took place online during the pandemic in comparison to the time before the pandemic and to determine whether their internal resource management strategies, intrinsic motivation, and preferences for instruction formats (synchronous, asynchronous, and face-to-face) affected how favorably they perceived their learning conditions.

### Digital learning environments in HE in times of COVID-19

In recent years, the increased capabilities of educational technology have expanded opportunities for online learning in HE (e.g., Wong et al., 2019). Although universities in many countries offer online instruction, digital learning environments have yet to be fully implemented in curricula. For example, a recent survey of HE in Germany found that just 1.7% of universities evaluated the digitalization of teaching and learning in their institution as being “well advanced” (Gilch et al., 2019). Recent analyses of the worldwide development of distance education revealed that Germany has made less progress in digital HE than countries with a pronounced tradition of distance education, such as Australia and Canada (Zawacki-Richter, 2020). Consequently, when the lockdowns began in response to the COVID-19 pandemic, many universities were not fully prepared for online teaching by the start of the summer 2020 term (Zawacki-Richter, 2020; Hertling et al., 2022).

In contrast to online learning environments, emergency remote teaching during COVID-19 was not accurately planned or well-designed and was instead characterized by a fast, temporary change to a different delivery mode of instruction (Hodges et al., 2020). This mode was viewed as a specific form of online instruction in which neither educators nor students participated voluntarily (Hodges et al., 2020; Naujoks et al., 2021).

The resulting learning spaces were classified as asynchronous or synchronous online courses (Ebner and Gegenfurtner, 2019; Alhazbi and Hasan, 2021). The synchronous mode enabled educators to interact with students in real time, while, in asynchronous courses, the educational material was available online, and student–teacher interactions were temporarily delayed to give students the opportunity to learn at their own pace (Öztok et al., 2013; Ebner and Gegenfurtner, 2019). Asynchronous courses offer high levels of flexibility but place heavy demands on the self-organization of students (Hratinski, 2008; Alhazbi and Hasan, 2021). Despite this rapid and sometimes ill-considered change, the possibilities of online teaching have been expanded and further developed and will continue to play a role in HE (Garcia-Morales et al., 2021). However, current research results show that students prefer face-to-face instruction over online courses (Abbasi et al., 2020; Aguilera-Hermida, 2020; Flores et al., 2021) and have difficulty with boredom, social isolation, time organization, and a lack of self-organizing capabilities (e.g., Liang et al., 2020; Mishra et al., 2020). In summary, the nature of this new digital learning environment obstructed the learning process in various ways and affected student learning experiences.

### Student perceptions of learning conditions

Based on models of learning and instruction, it is assumed that a student’s perception of their learning conditions is a relevant factor influencing their individual use of learning opportunities (Seidel and Prenzel, 2004; Gruber et al., 2010). According to Deci and Ryan (2002), feelings of competence and autonomy as well as social relatedness are the basic requirements of self-determined learning. Empirical results have also identified three conditions that play a role in student perceptions of supportive learning conditions: relevance of content, quality of instruction, and the enthusiasm of the teacher (Prenzel et al., 2002). Prenzel et al. (2002) summarized that students are supported in their needs if they perceive (a) a relevance of content, (b) instructional quality, (c) teacher enthusiasm in teaching, (d) social relatedness, (e) support of competence, and (f) support of autonomy. Research has shown that the perception of these supportive factors promote deeper processing and understanding of learning content (e.g., Artelt et al., 2003). Overall, these findings clearly point out the relevance of students’ perception of supportive learning conditions for their learning outcome.

This theoretical frameworks lend itself particularly well to investigating student perceptions of learning conditions, as the different aspects all relate to important characteristics of self-determined learning that takes place during distance learning (Holzer et al., 2021; Pelikan et al., 2021b; Ballad et al., 2022; Grande et al., 2022). For example, distance learning offers students greater possibilities with respect to organizing their learning, which may lead to increased perceived autonomy. At the same time, a lack of face-to-face contact may hamper feelings of social relatedness between students and their peers as well as students and their teachers.

In line with theoretical assumptions, recent research on student adaption to online teaching shows that, for students, the lack of support (i.e., access to the library and peer-support) and the lack of interaction were important challenges that arose during online learning (Aguilera-Hermida, 2020; Cavanaugh et al., 2022; Resch et al., 2022). Consistent with these results, other studies indicate that, at the end of the learning period, students reported negative experiences linked to relationships with their peers and technical difficulties (Karalis and Raikou, 2020; Lobos et al., 2022). Flores et al. (2021) found that students who felt less supported during distance learning, who rated the quality of instruction more poorly, or who received less feedback reported lower adaption to online learning. Furthermore, the study showed that students who preferred face-to-face formats over online teaching reported stronger feelings of fear and stress related to online education (Flores et al., 2021). Similarly, Händel et al. (2020) found that a student’s level of readiness for digital learning was related to feelings of stress and loneliness.

### The role of self-regulated learning and intrinsic motivation in distance education

Several different models of self-regulated learning (SRL) exist that describe how students take responsibility and regulate their own learning and performance (Boekaerts et al., 2000; Zimmerman, 2008). These theories commonly describe SRL as a constructive learning process wherein self-regulated learners use various cognitive and metacognitive strategies to control and regulate their learning (Zimmerman, 2000; Pintrich, 2004). In general, self-regulatory skills seem to be particularly important in online learning settings, as online learning is more flexible in terms of both time and participation (Lehmann et al., 2014). Online learning is typically less structured and SRL strategies are therefore considered to be even more important in distance learning than in traditional learning settings (Dabbagh and Kitsantas, 2004).

Thus, the correct use of SRL strategies can be considered essential to the academic outcomes of students during emergency remote learning (Deci and Ryan, 2002; Prenzel et al., 2002; Naujoks et al., 2021). The lack of in-class settings, limited face-to-face-interactions, and fewer support opportunities may require greater self-regulation and self-motivation (Littlejohn et al., 2016; Naujoks et al., 2021). In these situations, students suddenly need to plan, monitor, and control their learning processes more autonomously in order to follow self-study materials, organize participation in asynchronous and synchronous events, and communicate with peers and lecturers (Naujoks et al., 2021).

There are three key categories of learning strategies in SRL: cognition, metacognition, and resource management (Dresel et al., 2015; Panadero, 2017). Cognitive and metacognitive strategies are important for information processing and monitoring and for proving individual learning outcomes. Resource management consists of both external strategies (e.g., seeking help) and internal strategies like time management, motivation, and the regulation of effort and attention (Dresel et al., 2015).

In traditional in-class-face-to-face education, students with high self-regulatory abilities attain better academic achievements than those with lower self-regulation abilities (Mega et al., 2014; Dent and Koenka, 2015). Specifically, SRL is crucial in learning settings that provide low levels of support and guidance (Wong et al., 2019), especially distance-learning contexts (Zawacki-Richter, 2020; Naujoks et al., 2021). Prior studies have shown that SRL strategies are positively correlate with academic outcomes in online learning environments that afford high levels of learner autonomy (Broadbent and Poon, 2015; Broadbent, 2017). Specifically, internal resource-management strategies have been proven to play an essential role in the achievement of learning goals in online learning (Broadbent and Poon, 2015; Broadbent, 2017; Kizilcec et al., 2017; Grande et al., 2022). Thus, in situations where emergency remote learning, social distancing, and a variety of online learning applications are common, internal resource-management strategies may be key to successful autonomous learning characterized by pronounced reductions in social support (Biwer et al., 2021).

Current empirical studies support this assumption. Pelikan et al. (2021a) investigated how students coped with the challenges of distance learning during the COVID-19 pandemic and found that students with high self-perceived competence reported greater levels of intrinsic motivation and sophisticated learning strategies. However, the students who participated in this study also identified the existence of significant barriers to organizing their learning, keeping track of tasks, managing their time, and adhering to deadlines (Pelikan et al., 2021a).

Similarly, Biwer et al. (2021) examined university students’ adaption to emergency remote learning during the pandemic paying particular attention to resource management strategies. Their findings reveal that students experienced greater difficulties with time management and regulating their attention and efforts. In addition, students reported being less motivated by online learning than face-to-face instruction and also evaluated their general educational experiences as being of a lower quality (Biwer et al., 2021). Paetsch and Drechsel (2021) reported that attentional regulation predicted the perceived quality of teacher training and self-reported improvements in digital skills during distance learning. Finally, Naujoks et al. (2021) examined university students’ use of external resource management strategies (e.g., environment structuring, time management, and help-seeking) during distance learning and differences between students’ intended and actual use of these strategies. The results of this study showed that students felt technically prepared for online learning (e.g., they had access to necessary hardware and applications), but they did not apply as many resource regulation strategies as they intended to before entering the remote learning environment.

### Research questions and hypotheses

Distance learning requires greater self-regulation and intrinsic motivation for success compared to traditional learning settings (Broadbent and Poon, 2015; Broadbent, 2017; Kizilcec et al., 2017). Therefore, internal resource management strategies seem to be crucial for students when switching to emergency remote teaching (Naujoks et al., 2021). Students with pronounced internal resource management strategies and students with high intrinsic motivation should be more likely to adapt to the new situation. Based on models of learning and instruction, it is assumed that students’ perceptions of their learning conditions reflected their individual use of learning opportunities and refer to their learning success (Seidel and Prenzel, 2004; Gruber et al., 2010).

Therefore, the primary aim of this study was to investigate how HE students perceived their learning conditions during the first online semester compared to the time before the pandemic and determine whether internal resource management strategies and intrinsic motivation impacted their perceptions of changes in learning conditions. The second area of investigation was the role of instruction format (synchronous, asynchronous, and face-to-face) preferences in this context, as previous studies indicate that students’ format preferences relate to the feelings that they experience during online education (Flores et al., 2021). In this context, high preferences for digital instruction formats were viewed as an indicator of students’ readiness to engage in digital learning (Flores et al., 2021).

**Figure 1:**
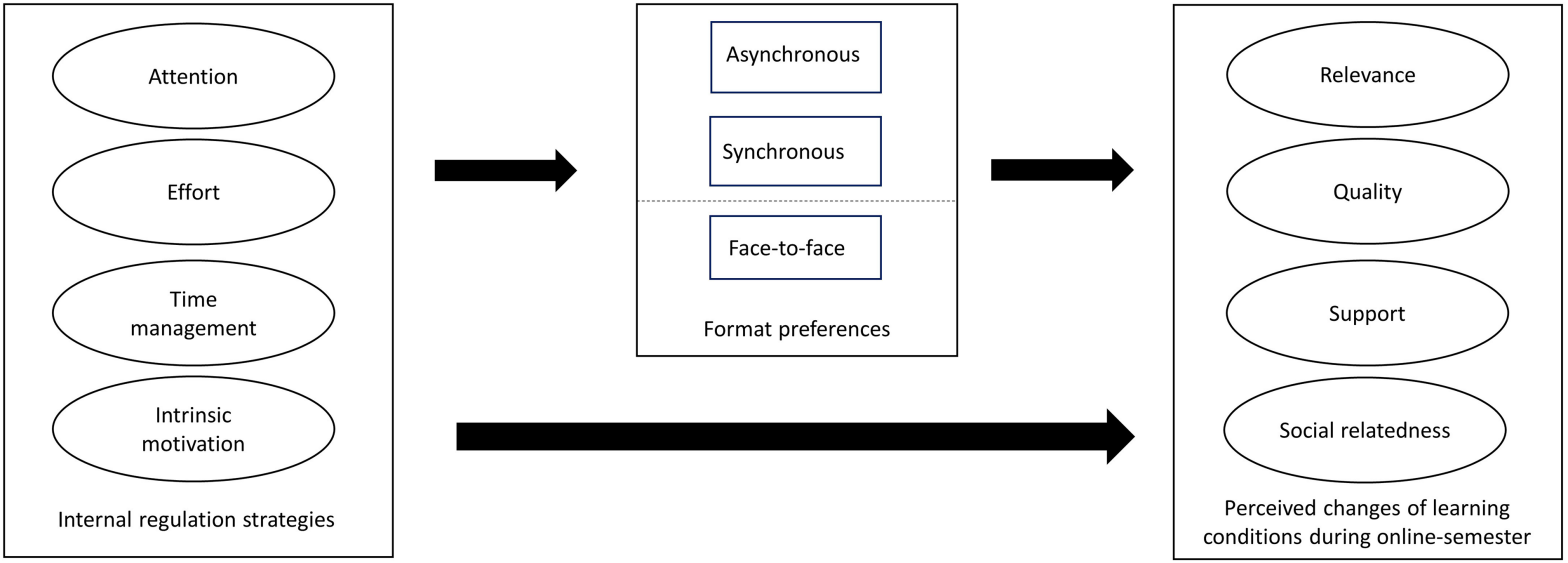
Hypothetical model.

Following the theoretical framework of student perception of supportive learning conditions (e.g., Ryan and Deci, 2000; Artelt et al., 2003; Prenzel et al., 2003; Seidel and Prenzel, 2004), this study focused on a learner-centered perspective that differentiates the central dimensions of learning conditions (see Figure 1). To examine the research questions, this study used data from the same sample as Paetsch and Drechsel (2021). In contrast to the previous study, this study investigated (a) different dimensions of learning conditions and (b) the role of instruction format preferences.

It was hypothesized that students’ perceived changes of learning conditions were predicted by their internal resource management strategies (effort regulation, time management, and attentional regulation), intrinsic motivation (*hypothesis 1*), and lesson format preferences. Because digital lesson format preferences indicate students’ readiness for digital learning, we expected positive effects relating to digital lesson format preferences and negative effects relating to face-to-face format preferences on students’ perceived changes of the learning conditions that they encountered during pandemic distance learning (*hypothesis 2*).

Because distance learning requires higher self-regulation and intrinsic motivation compared to face-to-face learning, it was also hypothesized that intrinsic motivation, effort regulation, time management, and attentional regulation would be positively associated with digital lesson-format preferences (*hypothesis 3*). Thus, students’ lesson format preferences mediated the relationship between students’ internal resource management strategies/intrinsic motivation and students’ perceived changes of the learning conditions (*hypothesis 4*). The hypothetical model is displayed in Figure 1.

## Materials and methods

### Sample

A total of 348 student teachers from a large public university in Germany participated in the present research study (see also Paetsch and Drechsel, 2021). This corresponds to 15% of all enrolled student teachers. For newly enrolled students, the measures of perceived changes of learning conditions were not meaningful because the time before distance learning was used as a benchmark in the questions. Therefore, 14 students declaring they were in their first semester of learning were excluded from the sample. Additionally, four participants with missing values for all variables were dropped from the sample. As a result, the final sample consisted of 330 student teachers (85% female, 14% male, and 1% non-binary). Such a high proportion of female students are typical in teacher education (Henoch et al., 2015). The participants studied different combinations of subjects and aimed to teach at a range of school types. Among the participants, 49% intended to work in elementary schools, 13% at secondary schools/middle schools, 22% at high schools/gymnasium, and 17% at vocational schools. The mean age of the participants was 22.5 years (*SD* = 3.1 years) with a mean study duration of 5.3 semesters (*SD* = 2.8 semesters).

Students were asked about how often have they participated in synchronous lectures (real-time teaching, i.e., Zoom) and asynchronous lectures (not limited to a specific point of time, i.e., video or audio recording) during the semester. The findings showed that 82.4% of the students had attended at ≥8 synchronous lectures and that 68.4% of the students had attended ≥8 asynchronous lectures. Students were also asked about their workloads. Most students (79.9%) reported that they spent >10 h in online classrooms, and 83.0% of students claimed that their general workload (including self-study) was higher than it had been during the previous (regular/non-pandemic) semester.

### Procedures

Data were collected through an online survey administered at the end of the first online semester in July 2020. The university provided online education throughout the whole semester with educators designing and organizing their courses autonomously. Our research study was announced, and the survey was distributed *via* websites, email lists, and social media. Student teachers completed an online questionnaire. The participation was voluntary, and participants were informed at a preliminary stage about the objectives of the investigation and how the data would be used in keeping with the ethical guidelines of human subject research. The confidentiality of the data and anonymity of participants were also assured.

### Measures

To take the circumstances of the pandemic into consideration, novel scales were developed to assess student perceptions of learning conditions in HE during the online semester and lesson format preferences. The process involved adapting several items from the scale created by Dalehefte and Munthe (2020). Each research instrument is described below. To verify the factor structure of observed variables, confirmatory factor analyses (CFA) were conducted.

#### Perceived changes of learning conditions during the online semester

To measure multifaceted learning conditions from a learner-centered perspective, 13 items were used that focused on the relevance of content, instructional quality, social relatedness, and support of competence and autonomy (Prenzel et al., 2002; Seidel and Prenzel, 2004). The time before distance learning was used as a benchmark in questions such as the following: *Compared to the time before the COVID-19 outbreak, how do you rate the average quality of instruction?* Respondents were asked to indicate the extent to which they agreed with each item on a five-point Likert-type scale (1 = much worse to 5 = much better). The reliability of all scales was acceptable to good: perceived relevance (three items, *α* = 0.79), perceived quality (three items, α = 0.72), perceived support (five items, *α* = 0.82), and perceived social relatedness (two items, *α* = 0.77).

#### Preferences for lesson format

With respect to teaching formats, a distinction was made between online formats (i.e., synchronous and asynchronous) and the traditional face-to-face format. These three formats were included in the measurement. The students reported the extent to which they favored each of the different lesson formats (synchronous, asynchronous, and face-to-face) on a five-point Likert-type scale based on the following question: *To what extent do you prefer the following types of instruction?*

#### Strategies for managing internal resources

The use of internal regulation strategies (attention, effort, and time management) was assessed using three scales from Klingsieck’s (2018) learning strategies of university students (LIST-K). All items were based on a five-point scale ranging from 1 (rarely) to 5 (very often). Attention (*α* = 0.89) was assessed based on three items (e.g., *While studying I’m easily distracted*). Effort (*α* = 0.63) was assessed using two items (e.g., *I do not give up even if the content is difficult and complex*). The original LIST-K effort scale contains three items; because of poor item characteristics one item was removed. Time management (*α* = 0.81) was assessed using three items (e.g., *While studying, I stick to a specific timetable*).

#### Intrinsic motivation

This study used the motivational regulation for learning in university students scale (SMR-LS) developed by Thomas et al. (2018). Specifically, the three-item intrinsic motivation scale of SMR-LS was used. *Currently, I enjoy studying* is an example of one of the items on the scale. Respondents were asked to indicate the extent to which they agreed with the items on a seven-point Likert-type scale (1 = very strongly disagree to 7 = very strongly agree). The reliability coefficient of the scale is *α* = 0.90.

### Statistical analysis

Data analysis was performed using IBM SPSS Statistics 26.0 (IBM Corp, 2017) and Mplus 8.7 (Muthén and Muthén, 2012). The percentage of missing values at the item level was low (maximum 7.6%). Data were missing completely at random based on Little’s (1988) MCAR test (χ^2^ = 533.98, *df* = 649, *p* = 1.00). To deal with the small number of missing values, the full information maximum likelihood approach (FIML) implemented in Mplus was employed. Robust maximum likelihood (MLR) estimation was the most appropriate fit for the Likert scales employed in the items. Significance testing was performed at the 0.05 level.

Confirmatory factor analyses were conducted to analyze construct validity, with two CFA models constructed for the eight latent variables (see Figure 1). The indicators of the latent variables were the items of the different scales. Structural equation modeling (SEM) was used to analyze the relationships of the hypothetical model. SEM is a multivariate quantitative technique used to estimate the relationships between observed variables and validate a theoretical model (Thakkar, 2020). Additionally, indirect effects on the four quality dimensions were investigated by decomposing the total effect into a set of direct and indirect effects.

Several indices were used to evaluate the model. We deployed the χ^2^/*df* test (<5), the root mean square error of approximation (RMSEA), the comparative fit index (CFI), the Tucker–Lewis index (TLI), and the standardized root mean square residual (SRMR). We adopted widely-used cutoff scores that reflect excellent and adequate fit to the data: TLI and CFI values above 0.95 or 0.90, RMSEA values below 0.06 or 0.08, and SRMR values below 0.08 (Hu and Bentler, 1999).

## Results

### Descriptive results and construct validity of scales

The descriptive results, correlations, and reliability scores of the constructs are presented in Table 1. The mean scores for intrinsic motivation (*M* = 4.61, *SD* = 1.32) and effort (*M* = 3.92, *SD* = 0.77) exceeded the midpoint of a five-point (three points) or seven-point scale (four points), indicating that students assessed themselves as being strong in these areas. The mean scores for attention (*M* = 2.58, *SD* = 1.04) and time management (*M* = 2.78, *SD* = 1.10), however, were below the midpoint, indicating less confidence in those areas. The mean scores for relevance (*M* = 2.81, *SD* = 0.75), quality (*M* = 2.89, *SD* = 0.77), and support (*M* = 2.74, *SD* = 0.73) were just below the midpoint, indicating that students considered pandemic distance learning conditions to be almost as good as before. Student perceptions of social relatedness (*M* = 1.89, *SD* = 0.87) during distance learning were clearly below the midpoint of the scale, indicating a noticeable decline. Results for format preferences revealed mean scores beyond the midpoint of the scale for all three formats with significant differences between the mean scores, as follows: synchronous (*M* = 3.37, *SD* = 1.06) < asynchronous [*M* = 3.67, *SD* = 1.24; *t*(322) = 3.52, *p* < 0.01] < face-to-face [*M* = 3.97, *SD* = 1.16; *t*(317) = 2.68, *p* < 0.01].

**Table 1:** Descriptive results, correlations, and reliabilities.

	**1**	**2**	**3**	**4**	**5**	**6**	**7**	**8**	**9**	**10**	**11**
1. Relevance											
2. Quality	0.71^**^										
3. Support	0.70^**^	0.62^**^									
4. Social relatedness	0.50^**^	0.41^**^	0.61^**^								
5. Asynchronous	0.42^**^	0.36^**^	0.36^**^	0.19^**^							
6. Synchronous	0.29^**^	0.26^**^	0.28^**^	0.08	0.09						
7. Face-to-face	−0.46^**^	−0.38^**^	−0.47^**^	−0.35^**^	−0.38^**^	−0.14^*^					
8. Intrinsic motivation	0.24^**^	0.24^**^	0.14^*^	0.10	0.13^*^	0.15^**^	0.09				
9. Internal regulation strategies: Attention	0.38^**^	0.36^**^	0.39^**^	0.22^**^	0.31^**^	0.22^**^	−0.35^**^	0.23^**^			
10. Internal regulation strategies: Effort	0.11^*^	0.15^**^	0.11	0.06	0.24^**^	0.00	−0.11	0.16^**^	0.35^**^		
11. Internal regulation strategies: Time management	0.08	0.10	0.09	0.07	0.19^**^	0.05	−0.12^*^	0.09	0.34^**^	0.31^**^	
Means	2.81	2.89	2.74	1.89	3.67	3.37	3.97	4.61	2.58	3.92	2.79
SD	0.75	0.77	0.73	0.87	1.24	1.05	1.16	1.32	1.04	0.77	1.10
Min	1.00	1.33	1.20	1.00	1.00	1.00	1.00	1.00	1.00	1.00	1.00
Max	5.00	5.00	5.00	5.00	5.00	5.00	5.00	7.00	5.00	5.00	5.00
Cronbach’s alpha	0.79	0.72	0.82	0.77	-	-	-	0.90	0.89	0.63	0.81
N	320	214	284	327	323	330	325	326	327	327	325
Missing values	10	16	46	3	7	0	5	4	3	3	5

** Correlation is significant at the 0.01 level (two-tailed).

* Correlation is significant at the 0.05 level (two-tailed).

Two separate CFAs were also conducted to confirm the factor structures of the latent variables. The first four-factor CFA model included 11 items measuring intrinsic motivation and the internal regulation strategies of attention, effort, and time management, respectively. The indices for this model indicated a good fit to the data (χ^2^ = 78.37, *df* = 38, *p* < 0.001, RMSEA = 0.06, SRMR = 0.04, TLI = 0.96, and CFI = 0.97) with factor loadings ranging from 0.61 to 0.93. This model showed a significant better model fit compared to the first-factor model using the Satorra–Bentler scaled chi-squared difference test (TRd = 609.21, *Δdf* = 6, *p* < 0.001). The second four-factor CFA model included 13 items measuring perceived relevance, support, quality of online instruction, and social relatedness during distance learning. Because modification indices revealed a correlation between errors of items 1 and 2 from the support scale, error correlation between them was allowed. The correlated items were more similar to each other than to the remaining items, as both addressed questions to the lecturer. The indices for this model also indicated a good fit with the data (χ^2^ = 112.28, *df* = 58, *p* < 0.001, RMSEA = 0.05, SRMR = 0.04, TLI = 0.95, and CFI = 0.97) with factor loadings ranging from 0.40 to 0.82. This model showed a significant better model fit compared to the first-factor model using the Satorra–Bentler scaled chi-squared difference test (TRd = 101.19, *Δdf* = 6, *p* < 0.001). The factor loadings for both CFA models are reported in Table 2. These results indicated that the construct validity of all scales was acceptable, and all of the latent variables were well-represented by the indicators.

**Table 2:** Standardized factor loadings for the items in the confirmatory factor analyses (CFA) models.

CFA models	Latent variable	Item	Factor loadings
Model 1 Internal regulation	Attention	1	0.84
		2	0.93
		3	0.81
	Effort^*^	1	0.75
		2	0.61
	Time management	1	0.76
		2	0.85
		3	0.69
	Intrinsic motivation	1	0.87
		2	0.90
		3	0.82
Model 2 Perceived learning conditions	Relevance	1	0.82
		2	0.64
		3	0.80
	Quality	1	0.63
		2	0.62
		3	0.81
	Support^**^	1	0.40
		2	0.61
		3	0.65
		4	0.79
		5	0.76
	Social relatedness	1	0.80
		2	0.78

* Because of poor item characteristics, we removed one item from the original learning strategies of university students (LIST-K) scale.

** Correlations between errors of items 1 and 2 from the support scale were allowed.

### Results of the structural equation modeling

The structural model was tested to examine the direct and indirect relationships between internal regulation strategies (attention, effort, and time management), intrinsic motivation, preferences for lesson formats, and perceived changes of learning conditions in HE during distance learning. The indices indicated an excellent fit for the model (χ^2^ = 428.112, *df* = 271, χ^2^/*df* = 1.58, RMSEA = 0.04 [0.034, 0.049], SRMR = 0.04, TLI = 0.94, and CFI = 0.96).

**Figure 2:**
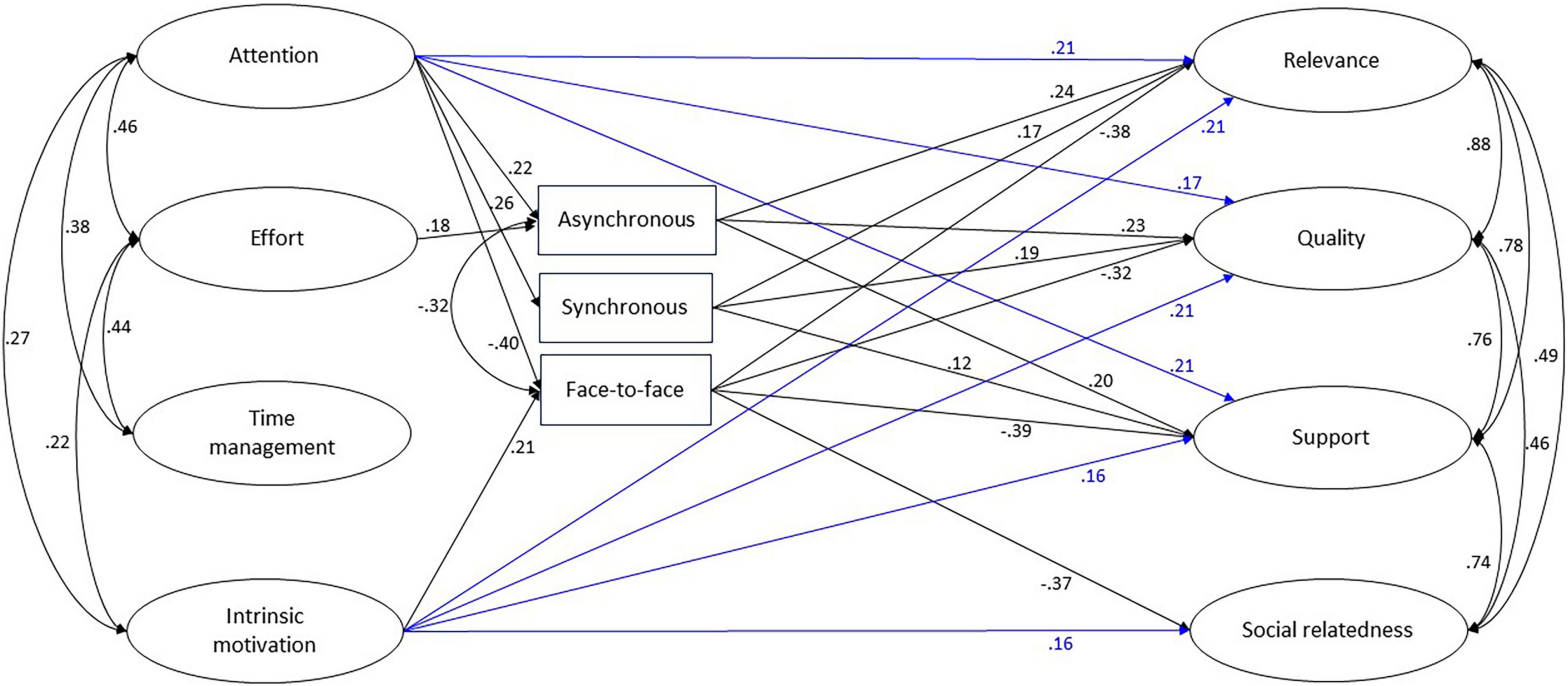
Structural equation model. Only paths *p* < 0.05 are displayed. See Table 3 for direct and indirect effects among variables. Blue = direct effects.

The findings reveal that attention and intrinsic motivation were significant predictors of perceived changes in relevance (β_ar_ = 0.21, *p* < 0.01; β_ir_ = 0.21, *p* < 0.01), quality (β_aq_ = 0.17, *p* = 0.02; β_iq_ = 0.21, *p* < 0.01), and support (β_as_ = 0.21, *p* < 0.01; β_is_ = 0.16, *p* = 0.02; see Figure 2). In other words, the more attention regulation or intrinsic motivation students had, the better they perceived the relevance, quality, and support of their courses compared to previous semesters. Significant direct effects of effort regulation and time management on perceived changes in relevance, quality, support, or social relatedness were not detectable. These results thus partially support hypothesis 1.

The relative preferences for asynchronous, synchronous, and face-to-face lesson formats were significant predictors of perceived changes in relevance (β_asr_ = 0.24, *p* < 0.01; β_sr_ = 0.17, *p* < 0.01; β_fr_ = −0.38, *p* < 0.01), quality (β_asq_ = 0.23, *p* < 0.01; β_sq_ = 0.19, *p* < 0.01, β_fq_ = −0.32, *p* < 0.01), and support (β_ass_ = 0.20, *p* < 0.01; β_ss_ = 0.12, *p* < 0.05; β_fr_ = −0.39, *p* < 0.01). As expected, all of the effects of face-to-face preferences on perceived changes of learning conditions were negative when taking preferences for the two digital formats under consideration. That means that the higher preferences for digital formats students had, the better they perceived the relevance, quality, and support of their courses compared to the time before the pandemic. In contrast, the higher preferences for face-to-face formats students had, the worse they perceived the relevance, quality, and support of their courses compared to the time before the pandemic.

For social relatedness, there was only a detectable significant negative association with preferences for face-to-face lesson formats (β_fs_ = −0.37, *p* < 0.01), indicating that students who rated face-to-face formats more positively reported less social relatedness during distance learning. These results support hypothesis 2.

The internal regulation strategies of attention, effort, and intrinsic motivation had significant effects on digital lesson format preferences (hypothesis 3). More specifically, attention predicted asynchronous preferences and synchronous preferences (β_aas_ = 0.22, *p* < 0.01; β_as_ = 0.26, *p* < 0.01); effort predicted only asynchronous preferences (β_eas_ = 0.18, *p* < 0.05). In other words, the more attention (effort) regulation students had, the higher their preferences for digital (asynchronous) formats were. Additionally, face-to-face format preferences were negatively predicted by attention (β_af_ = −0.40, *p* < 0.01) and positively predicted by intrinsic motivation (β_if_ = 0.21, *p* < 0.01). This result indicates that (a) students who had higher attention regulation reported less preference for face-to-face formats and (b) students who had higher intrinsic motivation reported greater preference for face-to-face formats. The internal regulation strategy of time management had no significant effects on students’ perceived changes in learning conditions or format preferences.

### Indirect effects on students’ perception of learning conditions during the online semester

The direct and indirect effects on student perceptions of learning conditions were estimated in Mplus using model indirect. Each specific indirect effect was calculated, and the significance of the mediation effects was tested with the Sobel test. The specific indirect effects and the bias-corrected bootstrap CIs are shown in Table 3. Of all the predictors, only attention had significant indirect effects on all dimensions of learning conditions (*z_r_* = 0.25, *p* < 0.01; *z_q_* = 0.23, *p* < 0.01; *z_s_* = 0.23, *p* < 0.01; *z_sr_* = 0.15, *p* < 0.01). Intrinsic motivation also had a small yet significant negative indirect effect on social relatedness (*z* = −0.08, *p* = 0.01). Thus, hypothesis 4 is supported only for attention. The direct relationships between attention and intrinsic motivation and relevance, quality, and support were statistically significant (see Figure 2; Table 3).

**Table 3:** Standardized indirect, direct, and total effects on students’ perceived changes of learning conditions.

Predictors	Effect	Relevance			Quality			Support			Social relatedness		
		Coefficient	95% CI^*^		Coefficient	95% CI^*^		Coefficient	95% CI^*^		Coefficient	95% CI^*^	
			Lower 2.5%	Upper 2.5%		Lower 2.5%	Upper 2.5%		Lower 2.5%	Upper 2.5%		Lower 2.5%	Upper 2.5%
Attention	Direct	**0.21** (<0.01)	0.05	0.34	**0.17** (0.02)	0.02	0.33	**0.21** (<0.01)	0.06	0.37	0.11 (0.17)	−0.04	0.29
	Indirect	**0.25** (<0.01)	0.17	0.34	**0.23** (<0.01)	0.16	0.31	**0.23** (<0.01)	0.15	0.32	**0.15** (<0.01)	0.05	0.25
	Total	**0.46** (<0.01)	0.30	0.60	**0.40** (<0.01)	0.24	0.56	**0.44** (<0.01)	0.29	0.59	**0.26** (<0.01)	0.10	0.44
Effort	Direct	−0.08 (0.34)	−0.29	0.09	−0.05 (0.57)	−0.25	0.12	−0.03 (0.75)	−0.21	0.14	−0.08 (0.40)	−0.27	0.12
	Indirect	0.03 (0.50)	−0.06	0.12	0.02 (0.56)	−0.06	0.11	0.03 (0.47)	−0.06	0.11	0.02 (0.55)	−0.06	0.09
	Total	−0.05 (0.57)	−0.27	0.13	−0.03 (0.77)	−0.23	0.16	0.01 (0.98)	−0.19	0.17	−0.06 (0.50)	−0.25	0.13
Time management	Direct	−0.07 (0.32)	−0.21	0.06	−0.03 (0.70)	−0.17	0.11	−0.01 (0.89)	−0.15	0.14	0.02 (0.77)	−0.15	0.18
	Indirect	0.01 (0.96)	−0.08	0.08	0.01 (0.93)	−0.07	0.08	0.00 (0.99)	−0.08	0.08	−0.01 (0.79)	−0.07	0.05
	Total	−0.06 (0.46)	−0.22	0.10	−0.02 (0.80)	−0.18	0.13	−0.01 (0.90)	−0.19	0.14	0.02 (0.84)	−0.16	0.18
Intrinsic motivation	Direct	**0.21** (<0.01)	0.10	0.34	**0.21** (<0.01)	0.11	0.36	**0.16** (0.02)	0.04	0.31	**0.16** (0.03)	0.03	0.32
	Indirect	−0.05 (0.21)	−0.12	0.03	−0.04 (0.32)	−0.10	0.04	−0.06 (0.10)	−0.13	0.02	**−0.08** (0.01)	−0.15	−0.02
	Total	**0.16** (0.03)	0.03	0.31	**0.18** (0.02)	0.04	0.33	0.10 (0.17)	−0.04	0.25	0.08 (0.27)	−0.07	0.22

* 95% bias-corrected bootstrap CI using 500 resamples.

Bold, significant at the 0.05 level (two-tailed).

## Discussion

Against the backdrop of the shift to online education necessitated by the COVID-19 pandemic, the present study aimed to shed light on the experiences of university students and to identify key factors that could predict student perceptions of learning conditions during pandemic distance learning. In Germany, digital learning had not been fully integrated into HE before the pandemic started (Gilch et al., 2019). Therefore, the rapid transition to online education in HE was characterized by improvised virtual classrooms with synchronous and asynchronous formats (Hodges et al., 2020) and thus might have been a challenge for students to navigate. Given the uniqueness of the emergency remote education and individual differences in self-regulated learning (Panadero, 2017), the success of adaption to the new situation may have varied among students.

Our descriptive findings indicate that, despite the sudden shift away from the traditional, classroom-based education format to a remote format, students believed their learning conditions in terms of relevance, quality, and support during the first online semester, on average, were equivalent to the conditions of traditional, pre-pandemic university instruction. However, students perceived a decline of social relatedness during distance learning compared to pre-pandemic times. This corroborates the findings of Hertling et al. (2022), who reported that students would have liked to have more interactive sessions and feedback from lecturers and peers.

Furthermore, our findings show significant differences between students’ preferences for lesson formats (asynchronous, synchronous, and face-to-face), with the highest mean preferences being for the face-to-face format and the lowest being for the synchronous format.

First, the present study investigated the relationship between internal resource management strategies (attention, effort, and time management), intrinsic motivation, and perceived changes in learning conditions during pandemic distance learning compared to the time before the pandemic at a large public university in Germany. Previous studies have shown that these are all important factors in online learning environments characterized by high levels of learner autonomy (Broadbent and Poon, 2015; Broadbent, 2017; Kizilcec et al., 2017). In our study, the regulative resources of attention and intrinsic motivation were found to be significant factors in the participants’ perception of changes in the relevance, quality, and support of online instruction, but they were not significant factors influencing perceived changes in social relatedness. As anticipated, students who reported higher levels of attention regulation and intrinsic motivation also evaluated the changes in relevance, quality, and support during online instruction as being more positive. This corroborates the findings of Biwer et al. (2021), who detected a positive correlation between attention and the educational experiences of HE students during the pandemic.

The findings of the current study indicated that students’ ability to concentrate and be attentive during learning without getting distracted and their learning enjoyment favored the adaption to the digital learning environment, which led to better experiences of quality instruction, relevance, and support. One possible explanation for the findings is the lack of external factors that support concentrated learning (e.g., in the classroom) and learning motivation (e.g., social recognition) during distance learning. Contrary to expectations, neither students’ regulation of effort nor time-management strategies predicted perceived changes of learning conditions. This result goes against the findings of Reinhold et al. (2021) and Biwer et al. (2021), who reported an increased need for self- and time-management after switching to distance learning (Reinhold et al., 2021) and positive associations between effort, time management, and educational experience during the first online semester (Biwer et al., 2021), respectively. However, in these studies, only bivariate correlations were examined, whereas our use of structural equation modeling provides a more nuanced explanation of the relationships among the variables. One possible explanation for the findings of the current study is that students did not notice increased time demands as much during this phase of the pandemic since the reduction in social interactions also freed up time resources for studying. This assumption aligns with the findings of Naujoks et al. (2021), who showed that time-management strategies were used less frequently during the online semester than students had previously intended. Zhang et al. (2021) also indicated that students succeeded in completing their assignments during the first online semester.

Another area of investigation focused on the role of lesson format preferences. In line with previous research (Aguilera-Hermida, 2020; Flores et al., 2021), students in this study preferred face-to-face instruction over online instruction formats. As expected, our results demonstrate that preferences for digital formats (synchronous and asynchronous) had notable and significant positive impacts on student perceptions of relevance, quality, and support, whereas preferences for the face-to-face format had significant negative effects on these factors. For social relatedness, we only observed a significant negative effect for the face-to-face preference, indicating that students who rated face-to-face formats more positively reported less social relatedness during distance learning. One possible explanation for this finding is that students who preferred face-to-face learning situations might have been less ready for digital learning, which led to difficulties in satisfying the need for social relatedness during distance education. This assumption is supported by empirical work showing the relationship between students’ readiness for digital learning and self-reported socio-emotions, such as fewer worries and reduced loneliness (Händel et al., 2020). This explanation also aligns with the findings of Flores et al. (2021), who showed that students’ face-to-face format preferences were associated with greater feelings of fear and stress during online teaching.

The final area of investigation focused on the indirect effects of internal resource management strategies and internal regulation on students’ perceived changes of learning conditions through preferences for lesson formats. Among the resource management strategies investigated in this study, only the regulation of attention was found to indirectly affect student perceptions of changes in relevance, quality, support, and social relatedness through lesson format preferences. These results indicate that high attention regulation fosters preferences for digital formats and hinders preferences for the face-to-face format, which leads to more positive perceptions of distanced learning conditions. In addition, students who showed more effort regulation reported higher asynchronous format preferences. Time management did not make any significant contribution to participants’ lesson format preferences, so there were no indirect effects on perceptions of learning conditions. One possible explanation for these findings is that students’ capabilities to concentrate, be attentive, and exert themselves during learning shape positive attitudes toward individualized learning processes with higher levels of autonomy, which is typical for digital learning environments (Means et al., 2013). Thus, students with more attention and effort regulation feel more comfortable with digital learning formats and show higher digital-format preferences compared to other students. The question of why internal strategies of time management were not related to format preferences remains open. Independent from their format preferences, all students were forced to study online during emergency remote teaching, which led to less adaption among students with high face-to-face format preferences.

Additionally, we found that intrinsic motivation had a small negative indirect effect on social relatedness through preferences for the face-to-face format. This means that students with high intrinsic motivation for learning in general had stronger preferences for the face-to-face format, which is associated with poorer perceptions of learning conditions during distance learning. One possible explanation for these findings is that intrinsic learning motivation refers, for some students, to traditional learning settings with face-to-face formats. These students could not profit from a high learning motivation during emergency remote teaching as they had a strong preference for the face-to-face format. However, there are substantial relationships between the three internal resource management strategies and intrinsic motivation. Hence, the findings further confirm the importance of internal resource management to successful online learning during the pandemic.

Overall, the current study contributes to the literature by emphasizing the important role that the internal resource management strategy of attention plays in HE distance education. In addition to the previous study (Paetsch and Drechsel, 2021), this study (a) revealed that the effects of resource management strategies differed across different dimensions of learning conditions and (b) uncovered the role of instruction format preferences in this context. Our findings about the negative effect of preferences for the face-to-face format support earlier research that highlights the connection between students’ readiness for digital learning and successful adaption to online learning (Händel et al., 2020; Flores et al., 2021). The consideration of different dimensions of learning condition perceptions reveals that social relatedness is not predicted by internal resource strategies but instead negatively predicted by preferences for the face-to-face format. A possible explanation for this finding could be that pandemic distance learning took place in a context with constricted social contacts in all areas of life and that even highly motivated and self-organized students were unable to adapt to the situation when it came to their need for social relatedness. The fact that intrinsic motivation was positively associated with preferences for the face-to-face format indicates that the satisfaction university students experience while learning is partially grounded on their basic need for social connectedness.

### Limitations and future directions

This study has several limitations. One of the limitations of this study is that the sample consisted of student teachers from only one German university who participated voluntarily. Therefore, the results were not representative of the student population in Germany. It seems possible that there is a relationship between participation in the survey and existing self-regulatory skills, in that less-structured learners may not have felt that they were able to participate in the survey. Future research could actively search for students with low self-regulatory skills during recruiting. Nevertheless, our sample included students from different subjects and types of school with varying study durations. Another limitation is that there were no *a priori* power analyses performed to specify the ideal sample size.

Furthermore, the validity of the newly developed instruments was not verified due to the novelty and rapidity of the situation. Retrospective self-report changes in learning conditions might differ from changes in perceptions of learning conditions. Further research is needed in this respect (e.g., longitudinal research designs, other measures to provide evidence of convergent validity).

Another limitation is the use of self-report instruments. There may be biases in the data due to socially desirable responses from participants, which could have led to results that differ from those obtained using other methods, such as behavioral observation. Student teachers’ self-reports were compared to those during a pre-pandemic baseline period, which means that the general level of perceptions of learning conditions was not considered. Moreover, multiple additional factors that influence perceptions of learning conditions remain uninvestigated (e.g., digital skills, students’ personal situations, access to technology, teachers’ behaviors, and personalities), but these factors lay beyond the scope of the current study. Future research on perceptions of learning conditions could include more aspects, such as students’ personal resources, prior experiences with digital learning environments, or professional knowledge, to generate deeper insights.

This study measures multifaceted learning conditions from a learner-centered perspective based on models of learning and instruction (Prenzel et al., 2002; Seidel and Prenzel, 2004), focusing on students’ perceptions of the relevance of content, instructional quality, social relatedness, and support of competence and autonomy. The perception of teacher enthusiasm, which is also an important factor in learning conditions (Prenzel et al., 2002), was not explored. Therefore, the results reflected only a limited picture of students’ perceptions of learning conditions. Future research on perceptions of learning conditions could include this factor to create a more widespread understanding of a learner-centered perspective. Moreover, this study focused on a learner-centered perspective and did not use other indicators for measuring the quality of the courses during the first online semester.

Another limitation is the cross-sectional study design. Therefore, the results are not informative for causal inference and identified relationships may not be easy to interpret; thus, bidirectional relations and reverse causality are possible. For example, positive experiences in autonomous learning during the pandemic might have improved intrinsic learning motivation or affected preferences for lesson formats.

Additional research is needed to examine the characteristics of digitalization in HE in non-pandemic contexts and their impact on student learning behavior under these conditions. Nonetheless, the results of the present study provide valuable information about the experiences of a group of students at a unique and highly challenging time and offer recommendations for improving HE practices.

### Implications for higher education and conclusion

Although the generalizability of our results is somewhat limited due to the cross-sectional nature of the study and the sampling method, this study offers insight into how student teachers experienced emergency remote teaching and the ways in which the sudden shift from a traditional classroom-based format to a digital format may have affected their perceptions of learning conditions. Although universities will transition back to face-to-face instruction after the pandemic, online learning and technology integration are likely to remain a part of HE.

Online learning settings differ from traditional HE settings in that the former require a greater degree of autonomous learning. Hence, fostering student resource management strategies seems to be a promising approach. Specifically, the present study sheds light on the importance of the internal regulation strategy of attention, which was directly and indirectly linked to students’ perceived changes of learning conditions. So, fostering internal resource strategies of attention should be made a priority in online teaching environments. Internal resource strategy attention can be supported in various ways, such as by offering stronger structuring and more guidance (Biwer et al., 2021) or through supporting students’ monitoring by asking prompting questions during online learning (Dignath and Büttner, 2008; Dresel and Haugwitz, 2008).

One crucial limitation of online instruction seems to be the restricted possibilities for social interactions with peers and faculty and extracurricular activities, which could result in weaker feelings of social relatedness, belonging, and social integration. The need for social relatedness is identified as a basic psychological need within social determination theory (Deci and Ryan, 2002). Therefore, enhancing social interaction in distance education by providing opportunities for communication in virtual learning groups may promote distance learning success (Broadbent and Poon, 2015) and facilitate students’ wellbeing (Deci and Ryan, 2002). In future online and hybrid learning environments, various methods of fostering social interaction should be developed, evaluated, and implemented. For this purpose, HE institutions could energize technology that enables authentic collaboration between students and offers possibilities to build communities within virtual spaces.

## Data availability statement

The datasets presented in this article are not readily available because informed consent signed by participants stated that data were only accessible to the authors of this study. Requests to access the datasets should be directed to JP, jennifer.paetsch@uni-bamberg.de.

## Ethics statement

Ethical review and approval was not required for the study on human participants in accordance with the local legislation and institutional requirements. The patients/participants provided their written informed consent to participate in this study.

## Author contributions

JP performed the statistical analyses and wrote the manuscript with support from AS. JP and AS discussed the results and contributed to the final manuscript. All authors contributed to the article and approved the submitted version.

## Funding

This project is part of the Qualitätsoffensive Lehrerbildung, a joint initiative of the Federal Government and the Länder which aims to improve the quality of teacher training. The program is funded by the Federal Ministry of Education and Research (01JA1915). This research was also funded by “Stiftung Innovation in der Hochschullehre” as part of the “Digitale Kulturen der Lehre entwickeln (DiKuLe)” project. The funders had no role in study design, data collection, analysis, decision to publish, or preparation of the manuscript. The authors are responsible for the content of this publication.

## Conflict of interest

The authors declare that the research was conducted in the absence of any commercial or financial relationships that could be construed as a potential conflict of interest.

## Publisher’s note

All claims expressed in this article are solely those of the authors and do not necessarily represent those of their affiliated organizations, or those of the publisher, the editors and the reviewers. Any product that may be evaluated in this article, or claim that may be made by its manufacturer, is not guaranteed or endorsed by the publisher.
